# ﻿A new species of the genus *Dryadaula* Meyrick (Lepidoptera, Dryadaulidae) from Japan, with a redescription of *D.epischista* (Meyrick, 1936)

**DOI:** 10.3897/zookeys.1217.122695

**Published:** 2024-11-12

**Authors:** Jinhyeong Park, Sadahisa Yagi, Shigeki Kobayashi, Toshiya Hirowatari

**Affiliations:** 1 Entomological Laboratory, Graduate School of Bioresource and Bioenvironmental Sciences, Kyushu University, 744 Motooka, Nishi-ku, Fukuoka, 819-0395, Japan Kyushu University Fukuoka Japan; 2 Entomological Laboratory, Faculty of Agriculture, Kyushu University, 744 Motooka, Nishi-ku, Fukuoka, 819-0395, Japan Osaka Metropolitan University Osaka Japan; 3 Insect Science and Creative Entomology Center, Kyushu University, 744 Motooka, Nishi-ku, Fukuoka, 819-0395, Japan Kyushu University Fukuoka Japan; 4 Environmental Entomology and Zoology, Graduate School of Agriculture, Osaka Metropolitan University, Sakai, Osaka, 599-8531, Japan Osaka Metropolitan University Osaka Japan

**Keywords:** Cytochrome *c* oxidase subunit I, molecular phylogeny, morphology, taxonomy

## Abstract

*Dryadaulaepischista* (Meyrick, 1936), initially described from a single male specimen in Japan, is herein redescribed based on newly collected specimens from the type locality. Furthermore, we describe *Dryadaulaorientalis* Park & Yagi, **sp. nov.**, a new species from Japan that closely resembles *D.epischista*. The adults and genitalia of the two species are illustrated. The genitalia of *D.epischista* from a specimen collected at the type locality are shown for the first time. DNA barcodes of the two *Dryadaula* species and the genetic distances of barcode regions among them and other congeners are provided.

## ﻿Introduction

The family Dryadaulidae was recently established for two genera, *Dryadaula* and *Brachydoxa*, based on molecular phylogenetic analysis, although these two genera were, in the past, included within Tineidae. Three genera, *Eschatotypa*, *Eugennaea*, and *Sagephora*, have been suggested to belong to Dryadaulidae based on asymmetrical male genitalia (Regier et al. 2015). However, details of the morphology, including wing venation, mouth parts, and female genitalia, have not been examined (Robinson and Nielsen 1993; Regier et al. 2015).

The genus *Dryadaula* currently has 50 described species (Yang and Li 2021; Lee et al. 2023) and has a pan-global distribution. Fourteen species occur in the Palearctic, 16 in the Neotropics, one in the Nearctic, two in the Afrotropics, four in Indomalaya, 12 in Australia, and two in Oceania, while *Dryadaulapactorlia* Meyrick, 1901, has been recorded in New Zealand and Europe (Yang and Li 2021; Lee et al. 2023). Only two species of *Dryadaula* species are described from Japan: *D.epischista* (Meyrick, 1936) and *D.trapezoides* (Meyrick, 1935) (Sakai 2013).

*Dryadaulaepischista* was described by Meyrick (1936) as *Thermocratesepischista* based on a single male specimen obtained in “Japan, Kyûsyû, Mozi (=Moji ward)”. Later, Robinson (1988) transferred it to the genus *Dryadaula* based on its genital morphology. The abdomen of the holotype was missing, but Robinson (1988) described the male genitalia of a specimen collected from Hong Kong as *D.epischista*. Before Zagulajev (1970), most species in this genus were described based only on external features. However, several species that are difficult to distinguish using external morphology were discovered recently, for example, *Dryadaulaauriformis* Yang & Li, 2021, *D.flavostriata* Yang & Li, 2021, *D.hirtiglobosa* Yang & Li, 2021, and *D.securiformis* Yang & Li, 2021. These facts suggest that the species diversity of this genus is underestimated, and that Hong Kong specimens identified as *D.epischista* by Robinson (1988), based on their appearance, were doubtfully conspecific to the holotype specimen from Moji, Fukuoka, Japan.

We conducted field surveys at the type locality to determine the true genital morphology of *D.epischista*. We successfully obtained specimens of *D.epischista* from the type locality and compared their morphology and DNA with those of specimens from other Japanese localities with similar appearances. In the course of the study, we observed that “*D.epischista*” from Hong Kong in Robinson (1988) is not representative of true *D.epischista* and discovered another unknown species from Japan. In this study, we describe a new species, *D.orientalis* Park & Yagi, sp. nov. and provide a redescription of *D.epischista* (Meyrick, 1936) with illustrations of adults and genitalia.

## ﻿Materials and methods

### ﻿Sampling and dissection

Specimens were collected by sweeping nets during the day and using an LED UV lamp (LTMW20S-BL, Iida shomei) at night (light trap: LT) and a Malaise trap. The material newly collected in this study is deposited in the Entomological Laboratory, Kyushu University (ELKU). We examined specimens deposited at the following universities and museums:

**NHMUK**The Natural History Museum, Department of Zoology, Cromwell Road, London SW7 5BD, United Kingdom;

**OMU**Environmental Entomology and Zoology, Osaka Metropolitan University (formerly Osaka Prefecture University, OPU), Sakai, Osaka, Japan;

**ELKU**Entomological Laboratory, Kyushu University, Fukuoka, Japan.

Images of adults were obtained using a SONY α7R IV digital camera (SONY, Tokyo, Japan) fitted with a CANON MP-E 65 mm macro lens (CANON, Tokyo, Japan). To examine male and female genitalia, the abdomens of specimens were removed and boiled in a 10% KOH solution for approximately 10 min. After washing with 70% ethanol, the genitalia were dissected in 70% ethanol and stained with a Chlorazol Black E solution. After dehydration by soaking in different concentrations of ethanol, from 70% to 99% for at least one hour, the genitalia were mounted in Euparal on glass slides. The specimens were dissected and observed under a Nikon stereomicroscope (SMZ-U). Images of genitalia were obtained using an EOS Kiss X5 digital camera connected to a Leica S8APO stereomicroscope and a Canon EOS 90D digital camera connected to a Nikon ECLIPSE Ci-L stereomicroscope. Photographs were processed using Adobe Photoshop 2022 and Adobe Photoshop 2023 processing software.

The terminology of genital morphology follows Robinson (1988).

### ﻿DNA extraction, PCR amplification, and sequencing

DNA analysis was performed to clarify male/female correspondence and genetic divergence in the partial mitochondrial cytochrome *c* oxidase subunit I (COI) region (DNA barcode region).

Adult specimens of *D.epischista* and *D.orientalis* sp. nov. were used for the extraction of DNA using a DNeasy Blood and Tissue Kit (Qiagen, the Netherlands). DNA was extracted from the abdomens of the moths using a kit protocol. To obtain the partial COI gene, the sequences were amplified using the primers LCO1490 (GGTCAACAAATCATAAAGATATTGG) and HCO2198 (TAAACTTCAGGGTGACCAAAAAATCA) (Folmer et al. 1994). The composition of the PCR reaction mixture used was as follows: 5 µL of KOD One® PCR Master Mix -Blue- (TOYOBO, Japan), 0.3 µL (10 pmol/µL) of each of the forward and reverse primers, and 1.0 µL of template DNA, with Milli-Q water added to a final volume of 10 µL. PCR amplification was performed using the following program: an initial denaturation at 98 °C for 10 s, followed by 35 cycles at 98 °C for 10 s, 50 °C for 5 s, and 68 °C for 5 s. The amplified products were purified using ExoSAP-IT™ Express (Thermo Fisher Scientific Inc., USA), and sequencing was conducted using the pre-mixed option at Sanger Sequencing Services (Azenta, USA). The alignment and deletion of ambiguous sites resulted in a COI of 658 bp.

Phylogenetic analyses were conducted using MEGA v.11.0.13 (Tamura et al. 2021). For constructing maximum likelihood (ML) trees and maximum parsimony (MP) trees for the partial COI mitochondrial genes, five *Dryadaula* species and two additional outgroup species [*Infurcitineacaptans* (Meessidae) and *Doleromorphaporphyria* (Tineidae)] were downloaded from the Bold Systems (Ratnasingham and Hebert 2007) and GenBank (Sayers et al. 2021). In summary, 17 OTUs, including specimens of *D.epischista* (two specimens) and *D.orientalis* sp. nov. (eight specimens), were analyzed using the General Time Reversible model. Branch support was calculated using 1000 bootstrap replicates. The uncorrected pairwise distances (p-distances) were calculated using *Dryadaula* species to construct the phylogenetic tree.

## ﻿Results

**Figure 1.**
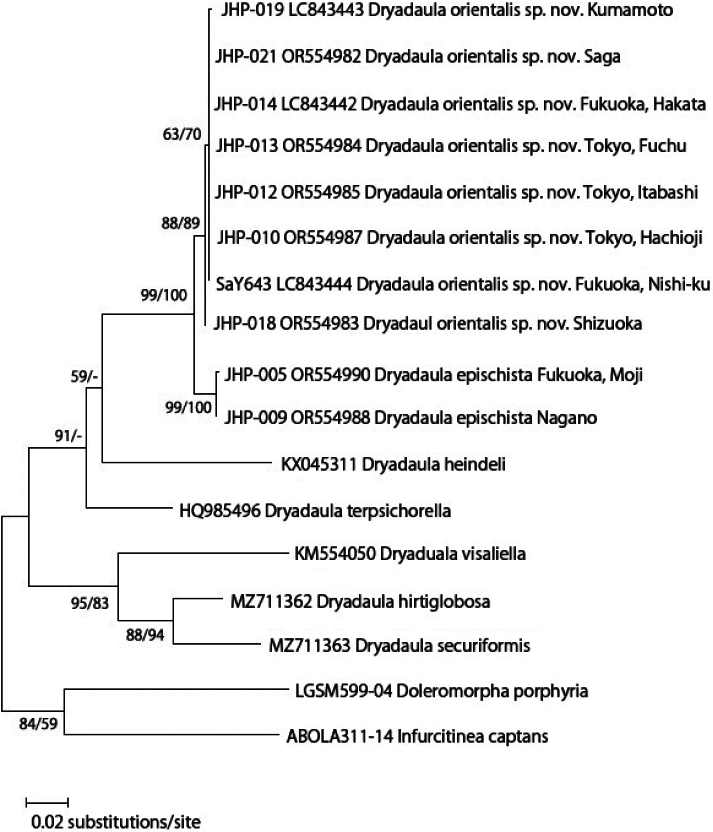
Maximum likelihood (ML) tree of *Dryadaula* constructed by MEGA 11 based on the partial COI region. The number near the node is the bootstrap value (ML/MP). Nodes annotated by “–“ indicate a mismatch in the topology between the ML and MP methods.

In the phylogenetic analysis, eight specimens of *D.orientalis* sp. nov. formed a sister group to two specimens of *D.epischista* (Meyrick, 1936) with high support (ML 99%, MP 100%; Fig. 1). The two Japanese species formed a single clade with *D.heindeli* Gaedike & Scholz, 1998 from Germany and *D.terpsichorella* (Busck, 1910) from the United States (bootstrap support = 91 in the ML analysis).

The results of pairwise distances (Table 2) indicated that interspecific distances between *D.epischista* (*N* = 2) and *D.orientalis* (*N* = 8) ranged from 1.52 to 1.83%, and intraspecific distances ranged from 0.15% in *D.epischista*, and 0.00 to 0.30% in *D.orientalis* sp. nov.

### ﻿Taxonomic accounts

#### ﻿ Dryadaula epischista

Taxon classificationAnimaliaLepidopteraDryadaulidae

(Meyrick, 1936)

30E944F1-0860-5400-ACEC-3A3994D45DD5

Figs 2 , 3 , 12 , 16 , 18–29 , 45–48

Thermocrates epischista Meyrick, 1936: 621.
Thermocrates epischista : Moriuti 1982, 1: 170, 2: pl. 233-2.
Dryadaula epischista : Robinson 1988: 70 (nec Meyrick 1936).
Dryadaula koreana Roh & Byun, 2020: Roh et al. 2020: 221. syn. nov.

##### Type material.

**Figures 2, 3.**
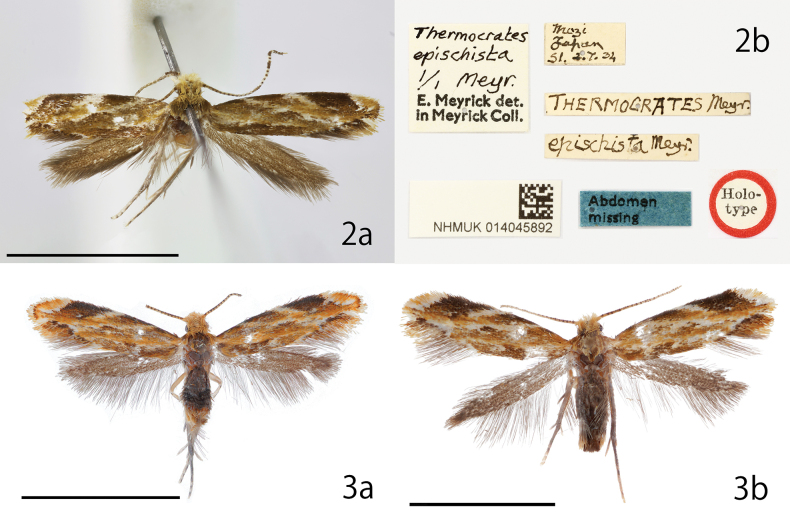
*Dryadaulaepischista* (Meyrick, 1936), adults **2a** holotype, male **2b** labels of holotype **3** Fukuoka, Moji, J.-H. Park leg. **3a** male, Museum ID ELKU-I-L-000043 **3b** female, ELKU-I-L-000044. Scale bars: 3 mm.

***Holotype***: Japan: • 1♂ (Fig. 2); Mozi [=Moji], Japan; 2. VII. 1934; S. Issiki leg.; *THERMOCRATES* Meyr.; *epischista* Meyr.; *Thermocratesepischista* 1/1 Meyr. E. Meyrick det. in Meyrick Coll.; abdomen missing; NHMUK 014045892; NHMUK.

##### Other material.

Japan: [Fukuoka] • 3♂; Kitakyushu, Moji, Mt. Tonoue-yama (33°54'00.2"N, 130°57'02.4"E); 153 m; LT (light trap); 22. VII. 2022; J.-H. Park leg. • 1♂ (Fig. 3a); same data; genitalia slide No. JP-025; DNA sample JHP-005; Museum ID ELKU-I-L-000043 • 1♂; same data; genitalia slide No. JP-044 • 1♂; same data; genitalia slide No. JP-023 • 4♂1♀; same locality; LT; 6. VIII. 2022; J.-H. Park leg. • 1♀ (Fig. 3b); same data; genitalia slide No. JP-051; DNA sample JHP-006; Museum ID ELKU-I-L-000044 • 1♂; same data; Museum ID ELKU-I-L-000042 • [Nagano] 5♂; Matsumoto, Arigase, Joyama Park (33°14'44.7"N, 137°57'15.2"E); LT; 5. IX. 2022; J.-H. Park leg. • 1♂; same data; DNA sample JHP-009; Museum ID ELKU-I-L-000045.

##### Diagnosis.

This species has an orange ground color on the forewing with a brown and white pattern and can be easily distinguished from other congeners by these characteristics. For the differences between *D.orientalis* sp. nov., which has a very similar color and forewing pattern, see Diagnosis of the latter species.

##### Redescription.

**Male** (Figs 2a, 3a): Forewing length 2.9 mm in holotype, 2.7–3.0 mm (*N* = 18); antenna length 1.6–2.1 mm (*N* = 9). Head small. Vertex and frons covered with yellowish-orange hair. Labial palpus spatulate, covered with cream yellow bristles; a few dark brown bristles arising laterally along second palpomere; third palpomere spatulate. Antenna filiform; scape and pedicel simple short columnar, flagellum dorsally covered with yellow scales, outer surface of flagellomere striped with blackish-brown and yellow, from the center to the tip, the blackish-brown parts fuse together to form four strips. Prothorax, mesothorax, and tegula covered with yellow and light-brown scales. Forewing costal and posterior margin straight, and gently curved toward apex. Forewing ground color orange, with brown and white pattern. Costa brown with three white lines at 1/4, 1/2 and apical part; the white line located at 1/2 of the costa connects to the tornal margin but is interrupted at center. Dorsum brown. Tornal area outer margin with brown line. Cilia orange, white basally; brown at tornus. Hind wing narrow trapezoidal, grayish brown. Abdomen covered with dark brown scales.

***Male genitalia.*** (Figs 12, 18–29, 45–48) Asymmetrical (Fig. 12). Uncus elongated and weakly curved to tip, and weakly twisted at subapically. Tegumen twisted to the left and slightly wider in the center, fused with vinculum. Vinculum narrowly arched; saccus equipped with obtuse triangle lobe at middle (Figs 18, 19). Gnathos absent. Right and left valva clearly asymmetrical (Figs 20–23, 29). Right valva flat; basal half with broad triangular lobe; apical half densely covered with relatively long setae; basally with small setose rod-shaped projection (Figs 20, 21). Left valva thick, but slenderer than right valva, tip paddle-like shaped, with lobate process and three spines: spines at the base short; spines on the left side of the center twice as long as spines at the base; spines on the right side of the center about the same length as spines on the left side, but they curve ventrally in the middle, in some cases, a single spine protrudes from the curved part; lobate process near apical part bearing spinose setae on dorsal surface (Figs 22, 23, 29). Sternite VIII hollow and curved claw- shaped; apical spine long and slender; ventral margin with two projections: central projection protruding trapezoidal, basal projection slender, longer than central projection (Figs 24, 25). Juxta rounded triangle and connected to left valva and phallus (Figs 12, 26). Phallus asymmetrical elliptical with two thick and sharp spines apically; rounded projection attached to juxta; cornuti absent (Figs 27, 28). Intersegmental membrane between seventh and eight tergite with a pair of free sclerites laterally; right free sclerite broad U-shaped, sometimes wider (Figs 45, 46); left free sclerite V-shaped (Figs 47, 48).

**Female** (Fig. 3b): Forewing length 2.8–3.2 mm (*N* = 3). Almost all same as male, but white spot of subapical part of costal margin narrowed and connected to white line of costal margin in female.

***Female genitalia*.** (Fig. 16) Sternite VIII covered with bristles, strongly sclerotized, right side markedly swollen posterolateral; terminal swelling weakly sclerotized and does not reach the ventral edge; ostium bursae opening the posterior end of swelling. Right side of “seventh segment” with ball-shaped bulge. Right side of “sixth segment” with posteriorly protruded bulge. Median dent deeply concave between right side of sixth and seventh segments. Papillae analis slender and short, 1.1× length of the apophyses posterioris. Apophysis posterioris relatively short, half the length of the eighth abdominal segment. Ductus bursae and corpus bursae membranous. Ductus bursae thin tubular, 5× as long as apophysis posterioris. Corpus bursae elliptical, densely wrinkled throughout (Fig. 16a).

##### Distribution.

Japan (Honshu, Kyushu), Korea.

##### Biology.

Unknown. Adults are observed from July to September.

##### DNA barcode.

Three DNA barcodes from two males and one female were generated and deposited in the International Nucleotide Sequence Database (INSD) and BOLD Systems (accession number/process ID): OR554990/JHP001-23, OR554989/JHP002-23, and OR554988/JHP003-23 (Table 1).

**Table 1.** Names and collection localities for taxa used in the phylogenetic analyses.

Species	Country	Site	Accession No. (sequence ID)	Sample ID
*Dryadaulaepischista*	Japan	Fukuoka, Moji	OR554990	JHP-005
*D.epischista*	Japan	Nagano, Matsumoto	OR554988	JHP-009
*D.orientalis* sp. nov.	Japan	Tokyo, Itabashi	OR554985	JHP-012
*D.orientalis* sp. nov.	Japan	Tokyo, Fuchu	OR554984	JHP-013
*D.orientalis* sp. nov.	Japan	Tokyo, Hachioji	OR554987	JHP-010
*D.orientalis* sp. nov.	Japan	Shizuoka	OR554983	JHP-018
*D.orientalis* sp. nov.	Japan	Fukuoka, Hakata	LC843442	JHP-014
*D.orientalis* sp. nov.	Japan	Fukuoka, Nishi-ku	LC843444	SaY643
*D.orientalis* sp. nov.	Japan	Saga, Arita	OR554982	JHP-021
*D.orientalis* sp. nov.	Japan	Kumamoto, Matsubase	LC843443	JHP-019
*D.heindeli*	Germany	Bavaria	KX045311	BC ZSM Lep 53186
*D.hirtiglobosa*	China	Zhejiang	MZ711362	DNAYLL18170
*D.securiformis*	China	Hainan	MZ711363	DNAYLL18122
*D.terpsichorella*	United States	Florida	HQ985496	BIOUG<CAN>: 10BBLEP -01124
*D.visaliella*	Canada	Ontario	KM554050	BIOUG03835-H04
*Doleromorphaporphyria*	United States	Tennessee	(LGSM599-04)	DNA-ATBI-0599
*Infurcitineacaptans*	Austria	Tyrol	(ABOLA311-14)	TLMF Lep 15333

**Table 2.** Genetic distances of the partial cytochrome *c* oxidase subunit I (COI) gene sequences of the two *Dryadaula* species. Genetic distances (%) were calculated with the p-distances model using MEGA 11.

	1	2	3	4	5	6	7	8	9	10
1. *Dryadaulaorientalis* sp. nov.										
JHP-010/OR554987/Tokyo, Hachioji										
2. *Dryadaulaorientalis* sp. nov.	0.00									
JHP-012/ OR554985/Tokyo, Itabashi										
3. *Dryadaulaorientalis* sp. nov.	0.00	0.00								
JHP-013/OR554984/Tokyo, Fuchu										
4. *Dryadaulaorientalis* sp. nov.	0.00	0.00	0.00							
JHP-014/LC843442/Fukuoka, Hakata										
6. *Dryadaulaorientalis* sp. nov.	0.00	0.00	0.00	0.00						
SaY643/LC843444/Fukuoka, Nishi-ku										
5. *Dryadaulaorientalis* sp. nov.	0.00	0.00	0.00	0.00	0.00					
JHP-021/OR554982/Saga										
7. *Dryadaulaorientalis* sp. nov.	0.15	0.15	0.15	0.15	0.15	0.15				
JHP-018/OR554983/Shizuoka										
8. *Dryadaulaorientalis* sp. nov.	0.15	0.15	0.15	0.15	0.15	0.15	0.30			
JHP-019/LC843443/Kumamoto										
9. *Dryadaulaepischista*	1.82	1.82	1.82	1.82	1.82	1.83	1.67	1.67		
JHP-005/OR554990/Fukuoka, Moji										
10. *Dryadaulaepischista*	1.67	1.67	1.67	1.67	1.67	1.68	1.52	1.52	0.15	
JHP-009/OR554988/Nagano										

##### Remarks.

Moriuti figured the “female” adult as the second specimen of this species; this specimen lacks the abdomen and the hindwings (Robinson 1988: 71). The specimen is not currently in OMU collection, missing.

The morphology of this species differs from that of the Hong Kong specimen identified as *D.epischista* by Robinson (1988) in the following characteristics: the left valva has three spines; the phallus has two apical spines; sternite VIII has longer elongated projections; and the basal half of the right valva is broadly triangular. However, we observed that *D.koreana* Roh & Byun, 2020 was identical to *D.epischista* based on its appearance and male genitalia. Therefore, here we synonymize *D.koreana* with *D.epischista*. We observed some variation in the apical spines protruding midway on the left valva occur in a few individuals within the same location.

#### ﻿ Dryadaula orientalis

Taxon classificationAnimaliaLepidopteraDryadaulidae

Park & Yagi sp. nov.

89115F0D-1DE4-59E3-81E5-E33F45F818E9

https://zoobank.org/18075FB2-38B0-41A3-8DC0-7A7CA03F50B0

Figs 4–11 , 13–15 , 17 , 30–44 , 49 , 50

Dryadaula epischista : Sakai 2013: 129, fig. 3-12-13 (nec Meyrick 1936) [examined]; Jinbo et al. 2014: fig. 8 (nec Meyrick 1936).

##### Type material.

**Figures 4–11.**
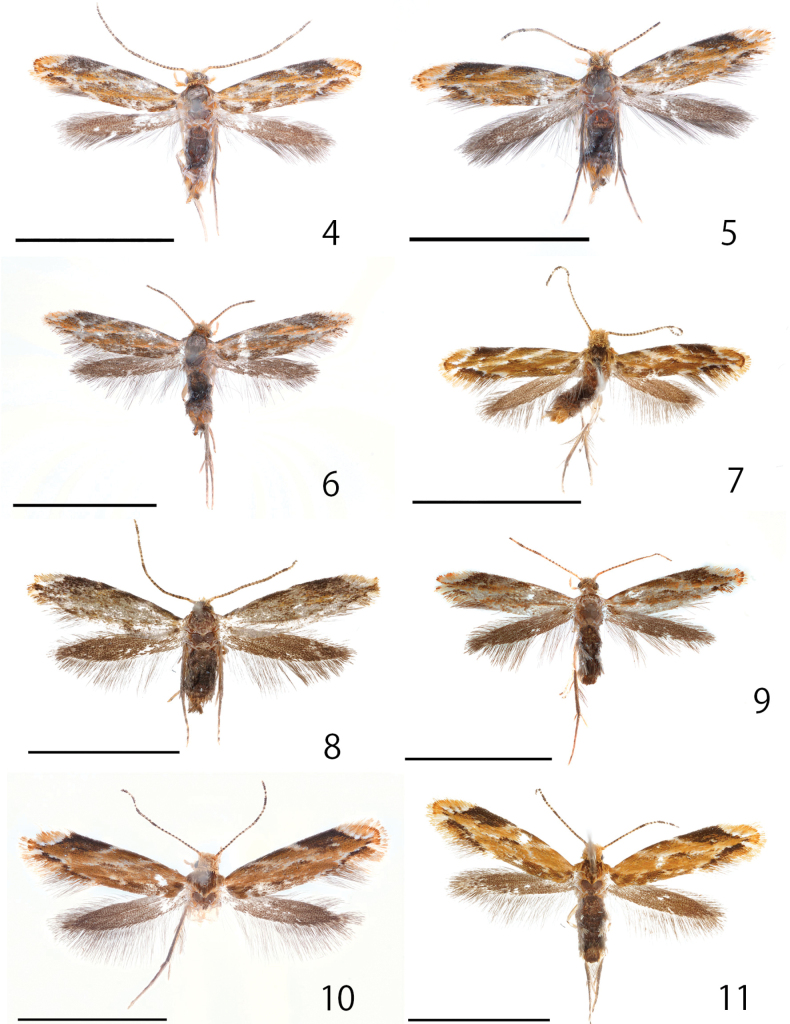
*Dryadaulaorientalis* sp. nov., adults **4** holotype, male, Tokyo, Itabashi, J.-H. Park leg., Museum ID ELKU-I-L-000048 **5–8** paratypes, male **5** Tokyo, Fuchu, J.-H. Park leg., ELKU-I-L-000049 **6** Tokyo, Hachioji, T. Hirowatari & J.-H. Park leg., ELKU-I-L-000046 **7** Ehime, J. Oku leg., ELKU-I-L-000130 **8** Saga, Yu Hisasue leg., ELKU-I-L-000051 **9–11** paratype, female **9** Tokyo, Hachioji, T. Hirowatari & J.-H. Park leg., ELKU-I-L-000041 **10** Fukuoka, Nishi-ku, S. Yagi leg., ELKU-I-L-000054 **11** Kumamoto, K. Goto leg., ELKU-I-L-000055. Scale bars: 3 mm.

***Holotype***: Japan: • 1♂ (Fig. 4); Tokyo, Itabashi, Akatsuka Park (35°47'05.4"N, 139°38'35.9"E); 26–27. VIII. 2022; J.-H. Park leg.; genitalia slide No. JP-028; DNA sample JHP-012; Museum ID ELKU-I-L-000048; deposited in ELKU. Materials were preserved in ELKU and OMU. ***Paratypes***: Japan: [Tokyo] • 1♂ (Fig. 5); Tokyo, Fuchu, Sengenyama Park (35°40'48.3"N, 139°30'01.5"E), 28. VIII. 2022; J.-H. Park leg.; genitalia slide No. JP-024; DNA sample JHP-013; Museum ID ELKU-I-L-000049 • 1♂1♀; Tokyo, Hachioji, Hatsuzawa, Mt. Hatsuzawa-yama; 1–2. X. 2022; J.-H. Park & T. Hirowatari leg.; (ELKU) • 1♀ (Fig. 9); same data; Museum ID ELKU-I-L-000041 • 1♂; same data; genitalia slide No. JP-047; DNA sample JHP-037; Museum ID ELKU-I-L-000052 • 1♀; same data; genitalia slide No. JP-039; DNA sample JHP-011; Museum ID ELKU-I-L-000047 • 1♂(Fig. 6); same data; DNA sample JHP-010; Museum ID ELKU-I-L-000046 • [Shizuoka] 1♂; Shizuoka, Kamo, Shimoda, Toji; 10. IX. 2022; LT; S. Yagi leg.; genitalia slide No. JP-035; DNA sample JHP-018; Museum ID ELKU-I-L-000050 • [Kyoto] 1♀; Kyoto, Higashiyama-ku, Seikanji, Yamanouchi-cho; 21. VI. 2014; H. SHIMIZU leg.; genitalia slide No. SK978; Museum ID OPU-IN-LE 2018XII0195; (OMU) • [Osaka] 1♀; Higashiosaka, Rokumanji-cho; 14. IX. 2014; H. SHIMIZU leg.; genitalia slide No. SK969; Museum ID OPU-IN-LE 2018XII0196; (OMU) • 1♀; Yao-shi, Kodachi Jusan Toge, Fumin no mori; 26. VIII. 2016; H. SHIMIZU leg.; genitalia slide No. SK979; Museum ID OPU-IN-LE 2018XII0197; (OMU) • 1♂; Hiraoka; 16. VI. 1995; S. KOSINO (leg.); Museum ID OPU-IN-LE 2018XII0193; (OMU) • 1♂; same data; genitalia slide No. SK968; Museum ID OPU-IN-LE 2018XII0194; (OMU) • [Ehime] 1♂ (Fig. 7); Matsuyama, Marunouchi, Matsuyama castle; 30. VIII. 2016; J. Oku leg.; genitalia slide No. JP-026; DNA sample JHP-022; Museum ID ELKU-I-L-000130 • [Fukuoka] 1♂; Fukuoka, Motooka, Kyushu Univ. (33.597N 130.214E); alt. 48 m; 4–18. IX. 2019; Malaise trap; Ent. Lab. Kyushu Univ. leg.; Museum ID ELKU-I-L-000040 • 1♂; Fukuoka, Hakata, Higashi-hirao Park; 19. VIII. 2022; LT; J.-H. Park leg.; genitalia slide No. JP-030; DNA sample JHP-014; Museum ID ELKU-I-L-000053 • 1♀ (Fig. 10); Fukuoka, Nishi-ku, Kuwabara, Kyushu Univ.; 1. VIII. 2020; S. Yagi leg.; genitalia slide No. JP-032; DNA sample SaY643; Museum ID ELKU-I-L-000054 • 1♂; same locality; 1. VIII. 2020; S. Yagi leg.; genitalia slide No. JP-027; DNA sample JHP-007; (ELKU) • [Saga] 1♂ (Fig. 8); Matsuura-gun, Arita-cho, Hiroseyama; 29. V. 2021; Yu Hisasue leg.; genitalia slide No. JP-029; DNA sample JHP-021; Museum ID ELKU-I-L-000051 • [Kumamoto] 1♀ (Fig. 11); Matsubase, Natl. Agricultural Res. Center; 6.VII.2022; K. Goto leg.; DNA sample JHP-019; Museum ID ELKU-I-L-000055.

##### Other materials.

[Fukuoka] • 1♂1♀; Fukuoka, Motooka, Kyushu Univ. (33.597°N, 130.214°E); alt. 48 m; 22. VIII–4. IX. 2019; Malaise trap; Ent. Lab. Kyushu Univ. leg; (ELKU) • 23♂26♀; Fukuoka, Motooka, Kyushu Univ. (33.597°N, 130.214°E); alt. 48 m; 4–18. IX. 2019; Malaise trap; Ent. Lab. Kyushu Univ. leg.; (ELKU) •1♂; same data; genitalia slide No. JP-042; (ELKU) • 1♀; same data; genitalia slide No. JP-034; (ELKU) • 1♀; (Fukuoka Pref.,) same locality (33.597°N, 130.214°E); alt. 48 m; 18. IX–2. X. 2019; Malaise trap; Ent. Lab. Kyushu Univ. leg.; (ELKU) • 1♀; (Fukuoka Pref.,) same locality (33.597°N, 130.214°E); alt. 48 m; 16–30. X. 2019; Malaise trap; Ent. Lab. Kyushu Univ. leg.; (ELKU).

**Figures 12–15.**
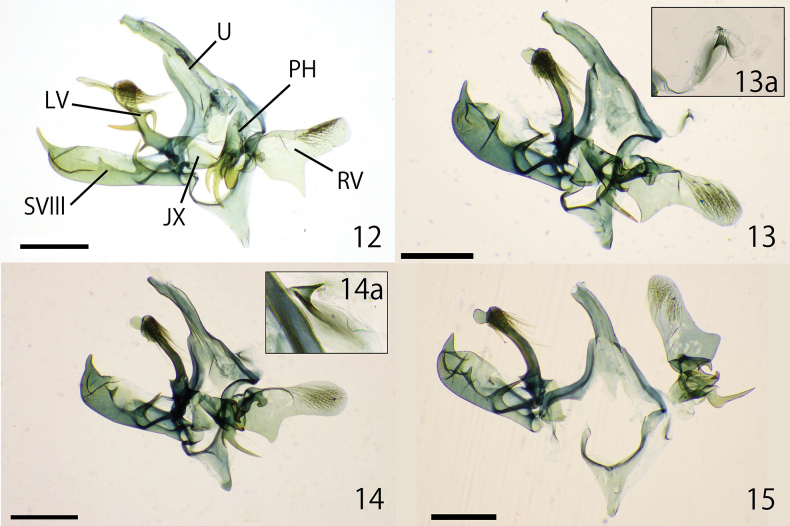
Overview of male genitalia **12***Dryadaulaepischista* (Meyrick, 1936), Fukuoka, Moji, J.-H. Park leg., genitalia slide No. JP-044; SVIII = sternite VIII; U = uncus; PH = phallus; JX = juxta; LV = left valva; RV = right valva **13–15***Dryadaulaorientalis* sp. nov. **13** holotype, Tokyo, Itabashi, J.-H. Park leg., genitalia slide No. JP-028 **13a** right free sclerite **14** paratype, Tokyo, Koganei, J.-H. Park leg., JP-024 **14a** right free sclerite **15** paratype, Fukuoka, Hakata, J.-H. Park leg., JP-030. Scale bars: 0.3 mm.

##### Diagnosis.

The color of the general habitus is very similar to *D.epischista*, but the new species can be distinguished by the following characteristics of the male and female genitalia: in the male genitalia, spines in the left valva are absent (present in *D.epischista*); in the female genitalia, a larger lateral abdominal swelling reaches the end of the abdomen in the eighth segment (smaller and not reaching the end of the abdomen in *D.epischista*).

##### Description.

**Male** (Figs 4–8): Forewing length 3.0 mm, antenna length 2.4 mm in holotype, Forewing length 2.7–3.7 mm (*N* = 11); antenna length 1.9–2.6 mm in paratypes (*N* = 9). Similar to *D.epischista* except forewing ground color varies from bright orange to dull brown.

***Male genitalia*** (Figs 13–15, 30–44, 49, 50) Asymmetrical (Figs 13–15). Uncus elongated and weakly curved to tip, and weakly twisted at middle. Tegumen twisted to the left and slightly wider in the center, fused with vinculum. Vinculum narrowly arched; saccus equipped with an obtuse triangle lobe at middle (Figs 30, 31). Gnathos absent. Right and left valva clearly asymmetrical (Figs 32–35). Right valva flat; basal half with broad triangular lobe, protrusions varied from sharp to rounded; apical half densely covered with relatively long setae; basally with small setose curved rod-shaped projection. (Figs 32, 33). Left valva thick, but slenderer than right valva, tip paddle-like shaped, with lobate process; lobate process near apical part bearing spinose setae on dorsal surface (Figs 34, 35). Sternite VIII hollow and curved claw-like shaped; apical spine short and thick; ventral margin with two slender projections, basal projection longer than central projection (Figs 36, 37). Juxta rounded triangle and connected to left valva and phallus (Figs 13–15, 38). Phallus asymmetrical elliptical with a curved, thick, and sharp spine apically; and with basal side of projection with or without a straight or curved thin projection; rounded projection attached to juxta; cornuti absent (Figs 39–44). Intersegmental membrane between seventh and eight tergite with a pair of free sclerites laterally; right free sclerite broad U-shaped (Figs 13a, 14a, 49); left free sclerite V-shaped (Fig. 50).

**Figures 16, 17.**
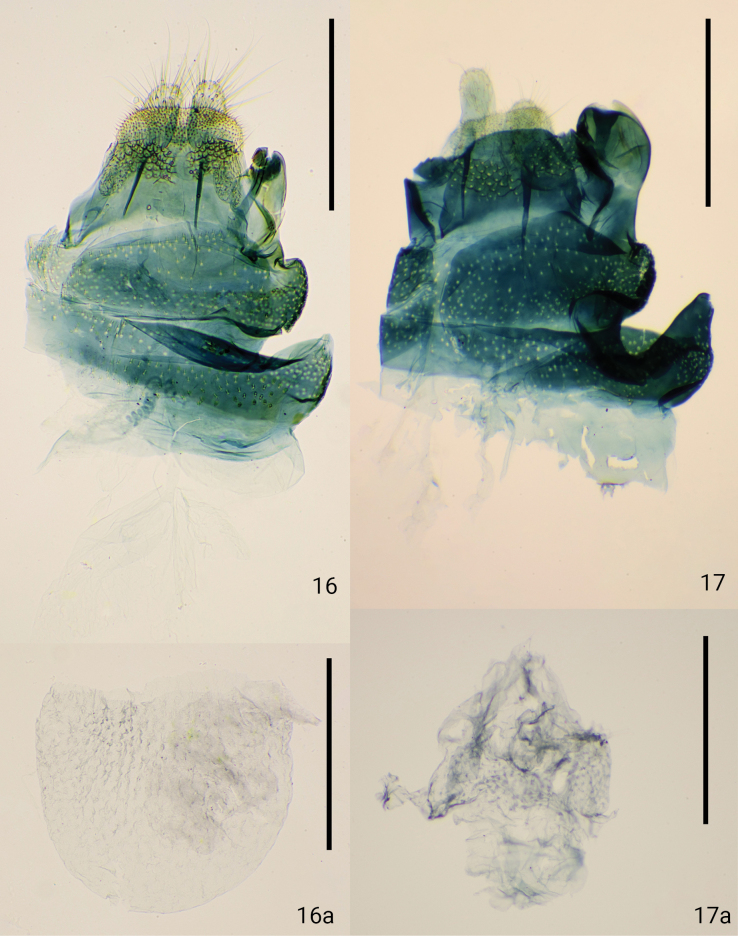
Overview of female genitalia, ventral view **16***Dryadaulaepischista* (Meyrick, 1936), Fukuoka, Moji, J.-H. Park leg., genitalia slide No. JP-051 **16a** corpus bursae **17***Dryadaulaorientalis* sp. nov., paratype, Tokyo, Hachioji, T. Hirowatari & J.-H. Park leg., JP-050 **17a** corpus bursae. Scale bars: 0.3 mm.

**Figures 18–29.**
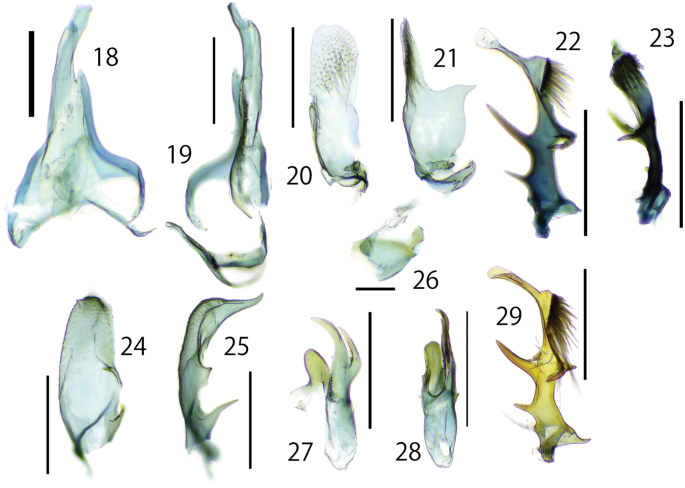
Details of the male genitalia of *Dryadaulaepischista* (Meyrick, 1936) **18**–**28** Fukuoka, Moji, J.-H. Park leg., genitalia slide No. JP-023 **18, 19** uncus, tegmen, vinculum **18** dorsal view **19** lateral view **20, 21** right valva **20** internal view **21** lateral view **22, 23** left valva **22** lateral view **23** dorsal view **24, 25** VIII sternites **24** lateral view **25** dorsal view **26** juxtae, dorsal view **27, 28** phallus **27** dorsal view **28** lateral view **29** left valva lateral view, Nagano, Matsumoto, J.-H. Park leg., Museum ID ELKU-I-L-000131. Scale bars: 0.3 mm (**18–25, 27–29**); 0.1 mm (**26**).

**Female** (Figs 9–11): Forewing length 3.0–3.6 mm in paratypes (*N* = 7), Antenna length 2.2–2.3 mm in paratypes (*N* = 4). Almost all same as male, but white spot of subapical part of costal margin narrowed and connected to white line of costal margin in female.

***Female genitalia*.** (Fig. 17) Sternite VIII covered with bristles, strongly sclerotized, right side markedly swollen posterolateral; terminal swelling passing the ventral edge; ostium bursae opening the posterior end of swelling. Right side of “seventh segment” slightly bulged. Right side of “sixth segment” with posteriorly protruded bulge. Median dent spherically and strongly concaves between right side of sixth and seventh segments. Papillae analis slender and short, same length as the apophyses posterioris. Apophysis posterioris relatively short, slightly longer than eighth abdominal segment. Ductus bursae and corpus bursae membranous. Ductus bursae thin tubular, 4× as long as apophysis posterioris. Corpus bursae elliptical, densely wrinkled from ductus bursae side to the end (Fig. 17a).

##### Distribution.

Japan (Honshu, Shikoku, Kyushu).

**Figures 30–44.**
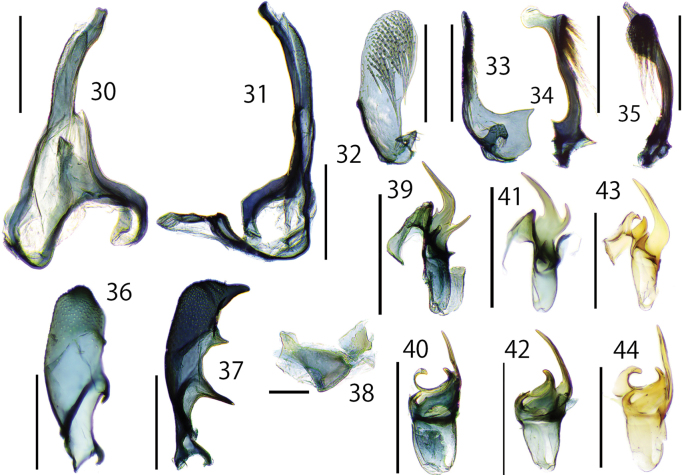
Details of male genitalia of *Dryadaulaorientalis* sp. nov. **30–40** Tokyo, Hachioji, T. Hirowatari & J.-H. Park leg., Museum ID ELKU-I-L-000046 **30, 31** uncus, tegmen, vinculum **30** dorsal view **31** lateral view **32, 33** right valva **32** internal view **33** lateral view **34, 35** left valva **34** lateral view **35** dorsal view **36, 37** sternite VIII **36** lateral view **37** dorsal view **38** juxta, dorsal view **39, 40** phallus **39** dorsal view **40** lateral view **41, 42** phallus, Fukuoka, Nishi-ku, Ent. Lab. Kyushu Univ. leg., ELKU-I-L-000040 **41** dorsal view **42** lateral view **43, 44** Osaka, Hiraoka, S. Kosino leg., Museum ID OPU-IN-LE 2018XII0194 **43** dorsal view **44** lateral view. Scale bars: 0.3 mm (**30–37, 39–44**); 0.1 mm (**38**).

##### Biology.

The adults were observed between May and September. Adult females laid flat eggs on the dead leaves of monocotyledonous plants (*Sasa* sp.) during plastic rearing.

##### Etymology.

The new species name is derived from the Latin ‘Orient’ (east) because the distribution of this species is restricted to eastern Asia.

##### DNA barcode.

Seven DNA barcodes from seven males and three females were generated and deposited in INSD and BOLD Systems (accession number/process ID (or sample ID)): OR554987/JHP004-23 (for holotype), OR55486/JHP005-23, OR554985/JHP006-23, OR554984/JHP007-23, OR554983/JHP008-23, OR554982/JHP009-23, OR554981/JHP010-23, LC843442/(JHP-014), LC843443/(JHP-019), LC843444/(SaY643) (for paratypes) (Table 1).

##### Remarks.

The phallus, right valva, and body color of this species exhibit marked geographical variation. Morphological variation was continuous, and there was little or no genetic distance between the specimens.

Many specimens of this species were collected in Malaise traps set at the forest margins in the forest of Kyushu University (26♂, 30♀).

## ﻿Discussion

The maximum likelihood tree suggested that *D.epischista* and *D.orientalis* sp. nov. were nested in a clade including *D.heindeli* and *D.terpsichorella*, but the former two species treated in this study differed in many characteristics, including narrow trapezoidal wings, flat right valva, and curved, twisted, and elongated uncus, whereas most species in this genus have broad trapezoidal hind wings. The South American species of this genus, *D.metrodoxa* (Meyrick 1919) and *D.amentata* (Meyrick, 1919), have narrow trapezoidal wings (Clarke, 1970: pl. 34, figs 1, 2), but DNA analysis has not been conducted, and additional research is needed to discuss their phylogenetic relationships in this genus.

**Figures 45–50.**
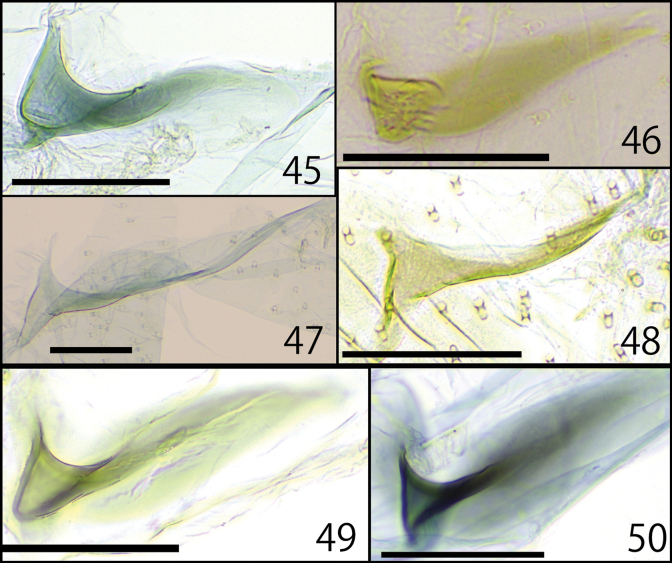
Free sclerites **45–48***D.epischista* (Meyrick, 1936) **45, 47** Fukuoka, Moji, J.-H. Park leg., genitalia slide No. JP-025 **45** right free sclerite **47** left free sclerite **46, 48** Nagano, Matsumoto, J.-H. Park leg., Museum ID ELKU-I-L-000045 **46** right free sclerite **48** left free sclerite **49, 50***D.orientalis* sp. nov. **49** right free sclerite, Fukuoka, Nishi-ku, Ent. Lab. Kyushu Univ. leg., JP-042 **50** left free sclerite, Tokyo, Hachioji, T. Hirowatari & J.-H. Park leg., ELKU-I-L-000046 Scale bars: 0.05 mm.

Robinson (1988) considered the free sclerites to be the abdominal seventh and/or eighth tergites. Free sclerites are a character only observed in *D.epischista* (Meyrick, 1936), *D.orientalis* sp. nov., and “*D.epischista*” from Hong Kong in Robinson (1988). The other external morphologies, including the genital characteristics of these species, are similar, and these species may be grouped together in future studies.

The results of this study indicate that the two closely related species are widely distributed in the southern part of Honshu and Kyushu but were never collected from the same locality (the closest point is approximately 50 km away in a straight line). The distribution of the two different species in geographically contiguous areas suggests that other factors, including host differences and interspecific competition, rather than geographic isolation, may influence the distribution of each species.

## Supplementary Material

XML Treatment for Dryadaula epischista

XML Treatment for Dryadaula orientalis

## ﻿Additional information

### Author ORCIDs

Jinhyeong Park https://orcid.org/0009-0005-3121-9990

Sadahisa Yagi https://orcid.org/0000-0002-4261-1219

Shigeki Kobayashi https://orcid.org/0000-0002-1336-4730

Toshiya Hirowatari https://orcid.org/0000-0002-6839-2229

### Data availability

All of the data that support the findings of this study are available in the main text.
